# Physicians Should Do Less Work

**Published:** 1896-10

**Authors:** 


### Physicians Should Do Less Work.

Dr. Kortright, in the Brooklyn *Medical Journal*, says that arterial sclerosis is a common cause of death of physicians. The lesson we should learn from our deceased colleagues, he states, is not to work too long. When you find your arterial tension increasing, your temporal artery becoming tortuous, your radial growing hard, especially if you have a little palpitation and pass an increased amount of limpid urine, whatever your years, know that old age is upon you. Henceforth shape your life like one that is old. Curb your ambition. Be content with a small practice. Reduce your expenses. Give up your night work. Decline confinements. Take a long vacation in summer. Retire early. Eat abstemiously. Drink not at all. Sell your horse. Take a great deal of moderate exercise in the open air. Watch the functions of the skin. Guard against a chill. Cultivate an even disposition. Study to be quiet.—*Maryland Med. Journal.*

### An Anomalous Condition.

An Irishman once told his physician "that he stuffed him with so many drugs that he was sick a long time after he got well."

